# Hydrogen Sulfide Promotes Thyroid Hormone Synthesis and Secretion by Upregulating Sirtuin-1

**DOI:** 10.3389/fphar.2022.838248

**Published:** 2022-02-10

**Authors:** Xue Zhao, Yedi Cao, Hongfang Jin, Xiuli Wang, Lanbo Zhang, Yang Zhang, Yang Yu, Youyuan Huang, Ying Gao, Junqing Zhang

**Affiliations:** ^1^ Department of Endocrinology, Peking University First Hospital, Beijing, China; ^2^ Department of Pediatrics, Peking University First Hospital, Beijing, China; ^3^ Department of General Surgery, Peking University First Hospital, Beijing, China

**Keywords:** hypothyroidism, hydrogen sulfide, sirtuin-1, thyroid hormone synthesis and secretion, thyrocytes

## Abstract

**Objective:** One mechanism of hypothyroidism involves the disruption of thyroid hormone synthesis and secretion by thyrocytes. Hydrogen sulfide (H_2_S), as a gas signaling molecule, participates in many physiopathologic processes by upregulating sirtuin-1 (SIRT1). The aim of the current study was to explore whether H_2_S promotes the synthesis and secretion of thyroid hormones by upregulating SIRT1.

**Methods:** Real-time PCR and immunohistochemistry were used to detect the mRNA and protein expression of H_2_S-generating enzymes in normal human thyroid tissues. Serum H_2_S concentrations from hypothyroid patients (*n* = 32) and euthyroid participants (*n* = 41) were detected by H_2_S-selective sensors. Thirty-one Sprague–Dawley rats were divided into control group (*n* = 10), hypothyroid group (induced by MMI, *n* = 10) and hypothyroid + NaHS group (*n* = 11), and the FT4, TT4 and TSH levels were assayed. Human primary thyrocytes were incubated with H_2_S donor sodium hydrosulfide (NaHS) or NaHS plus SIRT1 inhibitor (EX527) *in vitro*. Thyroid hormone synthesis- and secretion-related proteins [thyroid peroxidase (TPO), sodium iodide transporter (NIS), Pendrin, monocarboxylic acid transporter 8 (MCT8)] were analyzed by real-time PCR and Western blot.

**Results:** H_2_S levels in serum from hypothyroid patients were decreased compared to those from euthyroid participants (*p* < .05), and serum H_2_S levels were positively correlated with FT3, FT4, TT3, and TT4 levels in all subjects (all *p* < .0001). *In vivo*, NaHS promoted thyroid function in hypothyroid rats (*p* < .05). *In vitro*, H_2_S was detected in supernatant, and *CBS* mRNA was higher than *CSE* and *3-MPST* in human primary thyrocytes (*p* < .05). The protein levels of TPO, NIS, Pendrin and MCT8 were upregulated in a concentration-dependent manner for NaHS in thyrocytes. After blocking SIRT1 with EX527, we found that the increasing levels of TPO, NIS, Pendrin, and MCT8 and TPO activity were downregulated in thyrocytes incubated with NaHS, and FT4 levels in the cell supernatant were also decreased significantly (all *p* < .05).

**Conclusion:** H_2_S is mainly generated in thyrocytes by CBS. Serum H_2_S levels are decreased with hypothyroidism. H_2_S promotes the synthesis and secretion of thyroid hormones and the expression of related molecules by upregulating SIRT1.

## Introduction

Thyroid hormone plays an essential role in our body by regulating the function of physiological systems. Thyroid hormone synthesis and secretion in thyrocytes require related molecules, such as thyroid peroxidase (TPO) (Abramowicz et al., 1992), Pendrin (Taylor et al., 2002), sodium/iodide symporter (NIS) (Bagchi and Fawcett, 1973) and monocarboxylate transporter 8 (MCT8) (Di Cosmo et al., 2010). Hypothyroidism is a widespread endocrine disease that is characterized by reduced thyroid hormone production and secretion. Various diseases can be caused by hypothyroidism, such as heart failure (Biondi, 2012), hypertension (Tiller et al., 2016), reversible dementia (Muangpaisan et al., 2012). Based on a thyroid epidemiological survey published in China, the prevalence of hypothyroidism was 13.95% (Li et al., 2020). The specific mechanism of hypothyroidism is not completely clear. The most common physiopathologic mechanism of hypothyroidism is the irreversible destruction of thyrocytes (Giordano et al., 1997; Stassi and De Maria, 2002; Ralli et al., 2020). Currently, there has been no therapeutic approach to restore the function of destroyed thyrocytes, and long-term levothyroxine (L-T4) supplementation is an alternative therapy for hypothyroidism (Garber et al., 2012; Biondi and Wartofsky, 2014). Thus, it is critical to protect and maintain thyrocyte function in synthesizing and secreting thyroid hormones, which may be a new approach to hypothyroidism treatment.

Hydrogen sulfide (H_2_S) is the third most common endogenous gasotransmitter after carbon monoxide (CO) and nitric oxide (NO). H_2_S is produced endogenously in the body through both enzymatic and nonenzymatic pathways. Enzymes that can generate H_2_S mainly include cystathionine β-synthase (CBS) (Braunstein et al., 1969), cystathionine γ-lyase (CSE) (Chiku et al., 2009) and 3-mercaptosulfurtransferase (3-MPST) (Shibuya et al., 2009). H_2_S participates in multiple physiological and pathological processes by a variety of mechanisms (Mustafa et al., 2009; Sen et al., 2012; Untereiner et al., 2016; Wang et al., 2018). Several H_2_S-releasing drugs have been developed to safely treat a wide range of disorders, such as AP39 for oxidant-induced damage (Szczesny et al., 2014) and SG-1002 for heart failure (Elrod et al., 2007).

It was reported that CBS is increased in thyroid malignancies (Turbat-Herrera et al., 2018) and that H_2_S regulates the growth of different human thyroid carcinoma cell lines in a concentration-dependent manner (Wu et al., 2019). To the best of our knowledge, there has been almost no relevant research on H_2_S and thyroid function thus far. In the literature, H_2_S was found to participate in antiatherogenesis by S-sulfhydration of sirtuin-1 (SIRT1) (Du et al., 2019) and attenuate cellular senescence and apoptosis in alveolar epithelial cells by upregulating SIRT1 (Guan et al., 2019). Additionally, it was reported that high glucose exerted an effect on thyroid cell line damage and thyroid hormone deficiency through SIRT1 inactivation (Chen et al., 2019). Thus, we assumed that H_2_S might play a role in thyroid function by regulating SIRT1. The purpose of the current research was to study the change in H_2_S levels in hypothyroidism. Moreover, the effect of H_2_S on thyroid function and the specific mechanism were further explored.

## Materials and Methods

### Human Subjects and Samples

A total of 32 hypothyroid patients and 41 euthyroid participants were enrolled in this study. All euthyroid participants had no other thyroid diseases. None of the subjects had any infectious diseases, autoimmune diseases or other diseases that influenced serum H_2_S levels, such as acquired immune deficiency syndrome (AIDS), chronic hepatitis B, rheumatoid arthritis (RA), systemic lupus erythematosus (SLE), hypertension, atherosclerosis, asthma and inflammatory bowel disease (IBD). Any individuals with evidence of coexisting tumors were also excluded. Five hundred microliters of serum samples were stored at −80°C until use after collecting from all the subjects.

Serial sections of human thyroid tissues were collected from Peking University First Hospital. The samples were obtained from the contralateral lobe with papillary thyroid cancer (PTC), which was confirmed as normal thyroid tissues by pathologists. In addition, human thyroid tissues far away from the PTC lesion (*n* = 6) were collected for primary culture. All the patients were euthyroid and were diagnosed with no autoimmune thyroid diseases.

This research complied with the Helsinki Declaration and was approved by the Biomedical Research Ethics Committee of Peking University First Hospital.

### Animal Grouping and Preparation

To investigate the association between H_2_S levels and hypothyroidism *in vivo*, we established a hypothyroid model in rats and observed changes in thyroid function in these models after treatment with the H_2_S donor sodium hydrosulfide (NaHS). The protocols were approved by the Animal Research Ethics Committee of Peking University First Hospital.

Thirty-one male Sprague–Dawley rats (4 weeks old) were randomly divided into three groups: the control group (*n* = 10), hypothyroid group (*n* = 10) and hypothyroid + NaHS group (*n* = 11). After adaptive feeding for 1 week, the rats in the hypothyroid group were administered methimazole (MMI, 5 mg/100 g, Sigma–Aldrich, St. Louis, MO, United States) daily by intragastric administration for 3 weeks, while the rats in the control group were administered the same dose of saline intragastrically in the control group. The rats in the hypothyroid + NaHS group were injected intraperitoneally daily with NaHS (56 μmol/kg, Sigma–Aldrich) (Zhang et al., 2018) for 3 weeks, 2 hbefore a daily 5 mg/100 g MMI treatment, while the rats in the control and hypothyroid groups were injected with the same dose of saline intraperitoneally in the control and hypothyroid groups.

Furthermore, rats were anesthetized by ketamine after 3 weeks of saline, MMI or MMI + NaHS challenge. The bilateral total thyroid glands were removed rapidly, the weight was measured, and then the thyroid glands were stored in −80 °C or 10% neutral phosphate-buffered formalin. Serum samples from the rats in the three different groups were stored at −80°C until use.

### Thyroid Function Detection

Thyroid function in serum from the hypothyroid and euthyroid groups was assayed by chemiluminescence immunoassay (ADVIA Centaur; Siemens Healthcare Diagnostics, Camberley, UK). The detection range for free triiodothyronine (FT3): 3.50–6.50 pmol/L, free thyroxine (FT4): 11.48–22.70 pmol/L, total triiodothyronine (TT3): 0.92–2.79 nmol/L, total thyroxine (TT4): 58.1–140.60 nmol/L, thyroid-stimulating hormone (TSH): 0.55–4.78 μIU/ml. The lower detection limit for FT4 was 1.3 pmol/L.

Serum FT4 and T4 levels in three different groups of rats were detected by ELISA (CUSABIO, Wuhan, China), and TSH levels were measured by radioimmunoassay (FURUIRUNZE, Beijing, China). All procedures were conducted according to the protocols provided by the manufacturers. Intra-assay coefficients of variation were all less than 15%, and all serum samples were detected together to eliminate interassay variation.

### Measurement of H_2_S Levels

The H_2_S levels in serum samples from the subjects and rats were measured by using the free radical analyzer TBR4100 with an H_2_S -selective sensor (ISO-H_2_S-100, WPI, China) as reported previously (Huang et al., 2015). Firstly, the H_2_S-selective sensor was polarized with phosphate buffered saline (PBS) buffer, and then the calibration curve with a plot of the signal output (pA)–H_2_S concentration was generated as follows. The sensor tip was sequentially immersed in PBS buffer containing different concentrations of Na2S. Then, the calibration curve was completed by pA, which corresponded to the concentration of H_2_S. Second, the sensor tip was immersed into every sample to assay the H_2_S concentration according to the calibration curve.

### Histopathological Changes in Thyroid Tissues From Rats

Thyroid glands from rats in three different groups (control group, hypothyroid group and hypothyroid + NaHS group) were surgically removed and embedded in paraffin after fixing in 10% neutral phosphate-buffered formalin. Then, 5 μm thick sections were prepared for hematoxylin and eosin (H&E) staining to assess the pathological characteristics of thyroids from rats.

### Immunohistochemistry Staining (IHC) of Human Thyroid Slides for H_2_S Generation-Related Enzyme Detection

To test the expression of H_2_S-generating enzymes, including CBS, CSE and 3-MPST, in the human thyroid, the thyroid tissue sections mentioned above were microwaved in citrate buffer or EDTA buffer for 15 min, and then 3% hydrogen peroxide was used to block endogenous peroxidase. After blocking with 3% bovine serum albumin (BSA, Sigma–Aldrich), tissue slides were incubated with the following primary antibodies at 4°C overnight: CBS antibody (1:200), CSE antibody (1:25), and 3-MPST antibody (1:25) (all from Santa Cruz, California, United States). Then, the slides were incubated with secondary antibody for 60 min. Finally, 3,3′-diaminobenzidine (DAB) staining (ZSGB–BIO, Beijing, China) was used to detect positive areas, which were monitored by an Olympus BX51T microscope (Tokyo, Japan).

### The Isolation and Culture of Human Primary Thyrocytes

The thyrocyte culture method was performed on the basis of a method described previously (Nilsson et al., 1996; Zhao et al., 2018). The thyroid tissues were digested with type II collagenase (Gibco, Grand Island, New York, United States). After 1 h at 37°C, thyrocytes were digested with 0.05% trypsin (Gibco) for 5 min. After filtering through sterile filters, thyroid follicles were collected by centrifugation at 500 *g* for 3 min. Cells were cultured at 37 °C and 5% CO_2_ in RPMI 1640 medium containing 10% fetal bovine serum (FBS), 1% L-glutamine and 1% (v/v) penicillin/streptomycin (all from Gibco) supplemented with 1 mIU/ml bovine TSH (Sigma–Aldrich).

In addition, to detect FT4 levels in primary thyrocyte culture supernatant, thyrocytes were cultured with RPMI 1640 medium containing 1% charcoal-stripped fetal bovine serum (Biological Industries, Kibbutz Beit Haemek, Israel), 1% L-glutamine and 1% (v/v) penicillin/streptomycin (all from Gibco) and supplemented with 1 mIU/ml bovine TSH and 10^–8^ M sodium iodide (all from Sigma–Aldrich). H_2_S levels in primary thyrocyte culture supernatant were measured as described in Methods Section 2.4.

### Detection of H_2_S in Human Primary Thyrocytes by Fluorescent Probe *in situ*

Primary thyrocytes were cultured in Lab-Tek chambered cover glass (Thermo Fisher, Woolston, UK) and incubated with H_2_S fluorescent probes (1 μM, Cayman Chemical Company, Michigan, United States) for 30 min. Then, the cells were washed and fixed with 10% neutral phosphate-buffered formalin for 30 min. A TCS SP5 confocal laser-scanning microscope TCS SP5 (Leica, Wetzlar, Germany) was used to obtain fluorescent images.

### Human Thyrocyte Stimulation With NaHS and EX527 *in vitro*

To examine the effect of different concentrations of H_2_S on thyroid hormone synthesis and secretion-related molecules, thyrocytes were cultured as described in Methods Section 2.7. When the cell density reached approximately 60%, different concentrations of NaHS were added. Finally, the cells were collected after 48 h for Western blot to detect the changes in thyroid hormone synthesis- and secretion-related proteins.

To verify whether H_2_S exerted its effect on FT4 levels and TPO, Pendrin, NIS and MCT8 expression or TPO activity through SIRT1, thyrocytes were divided into three groups: the control group, NaHS group, and NaHS + EX527 group. The NaHS group was challenged with 100 μM NaHS. The NaHS + EX527 group was incubated with 10 μM EX527 (Sigma–Aldrich), a SIRT1 inhibitor, and 100 μM NaHS was added 4 h after EX527 treatment. Finally, the cells of all three groups were collected after 24 h for real-time PCR or 48 h for Western blot and TPO activity measurements, and the supernatant was also collected after 48 h for FT4 detection.

### Real-Time Polymerase Chain Reaction (Real-Time PCR) to Measure the mRNA Levels of Thyroid Hormone Synthesis- and Secretion-Related and H_2_S Generation-Related Molecules in Human Thyrocytes

TRIzol reagent (Life Technologies, Carlsbad, California, U.S.A.) was used to extract total RNA of human primary thyrocytes. The RNA was reverse transcribed into cDNA using the High Capacity cDNA Reverse Transcription Kit (Life Technologies). The primers for *TPO*, *NIS*, *Pendrin*, *MCT8*, *CBS*, *CSE*, *3-MPST*, and *GAPDH* are listed in Table 1. Detection of mRNA expression was carried out by the SYBR Green Supermix Kit (Thermo Fisher) and 7,500 real-time PCR system (Applied Biosystems, Foster City, CA, United States). Relative target gene expression was quantified by the 2−ΔΔCt method using *GAPDH* expression for normalization.

**TABLE 1:** Primer sequences used for real-time PCR.

Gene		Primer sequence (5’–3′)	Product bp
*TPO*	Forward	CTG​TCA​CGC​TGG​TTA​TGG​C	19
	Reverse	GCT​AGA​GAC​ACG​AGA​CTC​CTC​A	22
*Pendrin*	Forward	CAT​CAA​GAC​ATA​TCT​CAG​TTG​GAC​CT	26
	Reverse	ACA​GTT​CCA​TTG​CTG​CTG​GAT	21
*NIS*	Forward	GTC​CTT​CAG​GGC​TCC​TTC​ACC	21
	Reverse	CTG​CTC​GCT​GGG​TGG​GTA​CA	20
*MCT8*	Forward	CCA​CGC​CTA​CGG​TAG​AGA​C	19
	Reverse	CAG​AGT​TAT​GGA​TGC​CGA​AGA​TG	23
*CBS*	Forward	AATGGTGACGCTTGGGAA	18
	Reverse	TGAGGCGGATCTGTTTGA	18
*CSE*	Forward	AAG​ACG​CCT​CCT​CAC​AAG​GT	20
	Reverse	ATA​TTC​AAA​ACC​CGA​GTG​CTG​G	22
*3-MPST*	Forward	CGG​AGT​CTC​CTC​CCT​TTG​GT	20
	Reverse	CCT​CCC​TAA​GAT​GCA​GCT​CG	20
*GAPDH*	Forward	GGA​GCG​AGA​TCC​CTC​CAA​AAT	21
	Reverse	GGC​TGT​TGT​CAT​ACT​TCT​CAT​GG	23

### Western Blot Analysis of the Protein Levels of Thyroid Hormone Synthesis- and Secretion-Related Molecules in Human Thyrocytes

Primary thyrocyte lysates (25 μg per sample) were separated by sodium dodecyl sulfate-polyacrylamide gel (10%) electrophoresis and spotted onto nitrocellulose (NC) membranes. The NC bands were separately incubated with the following primary antibodies at 4°C overnight: NIS antibody (1:2000; Biorbyt, Cambridge, UK), TPO antibody (1:400; Santa Cruz, California, United States), Pendrin antibody (1:500; Abcam, Cambridge, UK), MCT8 antibody (1:500; Proteintech, Wuhan, China), SIRT1 antibody (1:500, Abcam), and GAPDH antibody (1:2000; TransGen Biotech, Beijing, China). The bands were then incubated with secondary antibodies (1:5000; ZSGB–BIO) for 1 h. The immunoreactions were detected by an enhanced chemiluminescence system (Millipore, Yonezawa, Japan). ImageJ software (developed at the National Institutes of Health) was used to quantify the relative staining intensity.

### Enzymatic Activity of TPO in Human Primary Thyrocytes

We assayed TPO activity as previously described (Godlewska et al., 2014). The cells were washed three times. Then, 500 µL of reaction mixture was added [100 μM potassium iodide (Sigma–Aldrich), 50 μM Amplex Red (Life Technologies) and 200 U/ml superoxide dismutase (Sigma–Aldrich)]. The reaction was started via adding hydrogen peroxide (Life Technologies). Twenty-microliter aliquots were mixed with 80 µL inhibition mixture [500 U/ml catalase (Solaria, Beijing, China) and 100 U/ml SOD (Sigma–Aldrich)] after removing every minute for 8 min. The fluorescence was assayed in a microtiter reader (Bio Tek, Vermont, United States) by using excitation at 530 nm and emission at 590 nm. The detection values were corrected by the corresponding protein quality.

### Statistical Analysis

All experimental data were analyzed by using SPSS 25.0 (IBM, United States). Normally distributed variables are expressed as the mean ± standard deviation (SD) or mean ± standard error (SEM), and nonnormally distributed variables are expressed as the median and interquartile range. Means were compared via one-way ANOVA for comparisons among multiple groups and independent t tests for comparisons between the two groups. Medians were compared by Kruskal–Wallis test among multiple groups and Mann–Whitney test between the two groups. Bivariate relationships were performed with a Pearson or Spearman rank correlation model. *p* < .05 was considered as statistically significant.

## Results

### Human Primary Thyrocytes Produced H_2_S, and CBS Was the Main H_2_S-Generating Enzyme in Normal Thyrocytes

**FIGURE 1:**
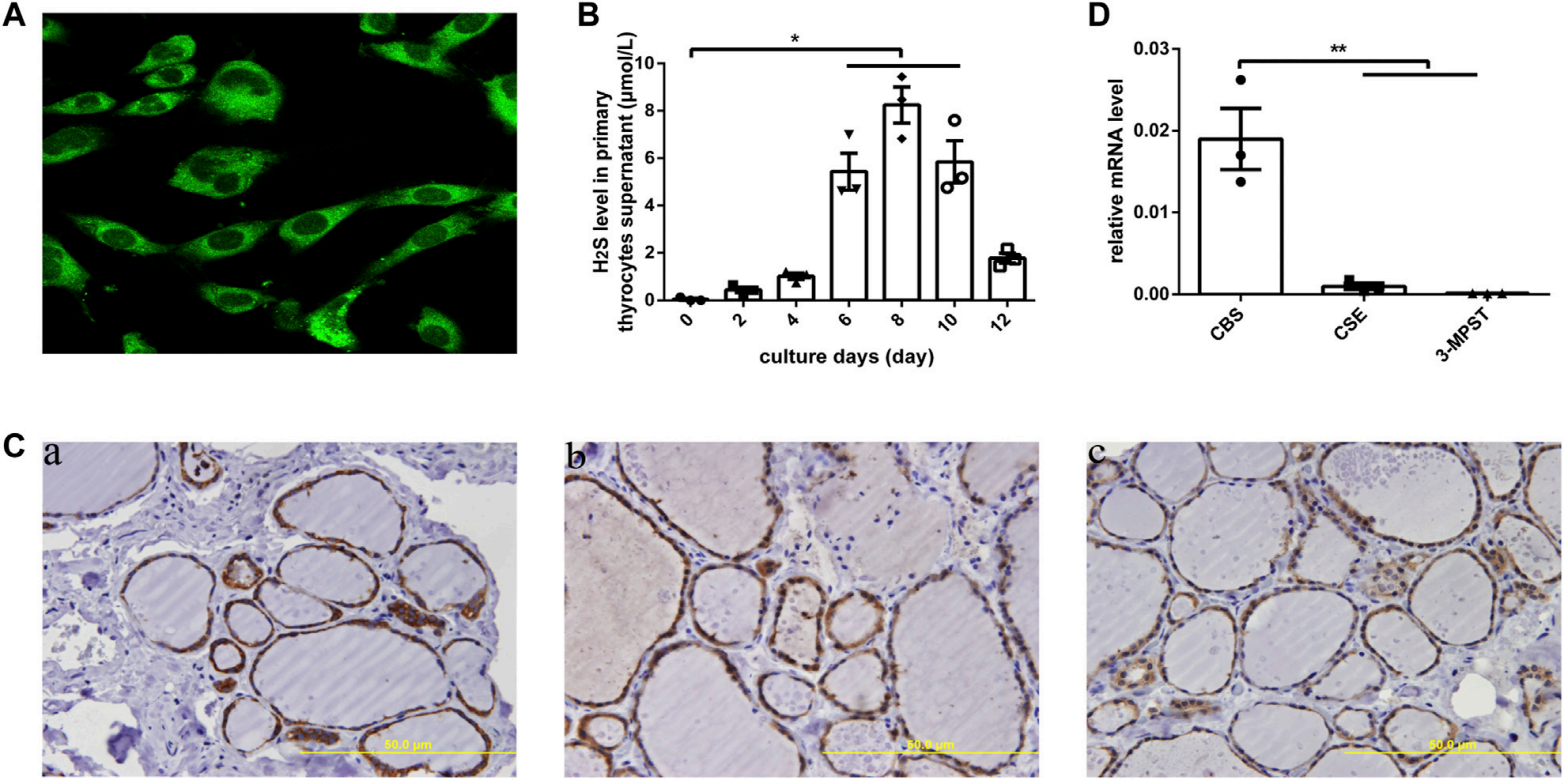
H_2_S generation and related enzymes in human thyroid. **(A)** H_2_S generation in human primary thyrocytes was measured by an *in situ* fluorescent H_2_S probe. **(B)** H_2_S levels in supernatants were detected by H_2_S-selective sensor in human primary thyrocytes with different culture days. **(C)** The enzymes that generated H_2_S were detected by immunohistochemistry staining in human thyroid tissues. All of them were located in the cytoplasm of thyrocytes. CBS (a), CSE (b), 3-MPST (c). **(D)** The enzymes that generated H_2_S in human primary thyrocytes were detected by real-time PCR. The level of *CBS* mRNA expression is the highest. *p < .05, **p < .01. CBS, cystathionine β-synthase; CSE, cystathionine *γ*-lyase; 3-MPST, 3-mercaptosulfurtransferase; H_2_S, hydrogen sulfide. Data are expressed as the mean ± SEM, and all experiments were performed independently three times.

In human primary thyrocytes, we measured H_2_S production by an *in situ* fluorescent probe (Figure 1A), and at different culture days, H_2_S was also detected in supernatants by a H_2_S-selective sensor (Figure 1B), which indicated the capacity of thyrocytes to produce H_2_S. We further assayed H_2_S-generating enzymes in human thyroid tissues by IHC and found that CBS, CSE, and 3-MPST all existed in thyrocytes and were mainly localized in the cytoplasm (Figure 1C). Then, we observed significantly higher mRNA expression for *CBS* than *CSE* and *3-MPST* in human primary thyrocytes, which illustrated that CBS is the main H_2_S-generating enzyme in the thyroid (Figure 1D).

### Decreased H_2_S Content in Serum From Hypothyroid Patients

**FIGURE 2:**
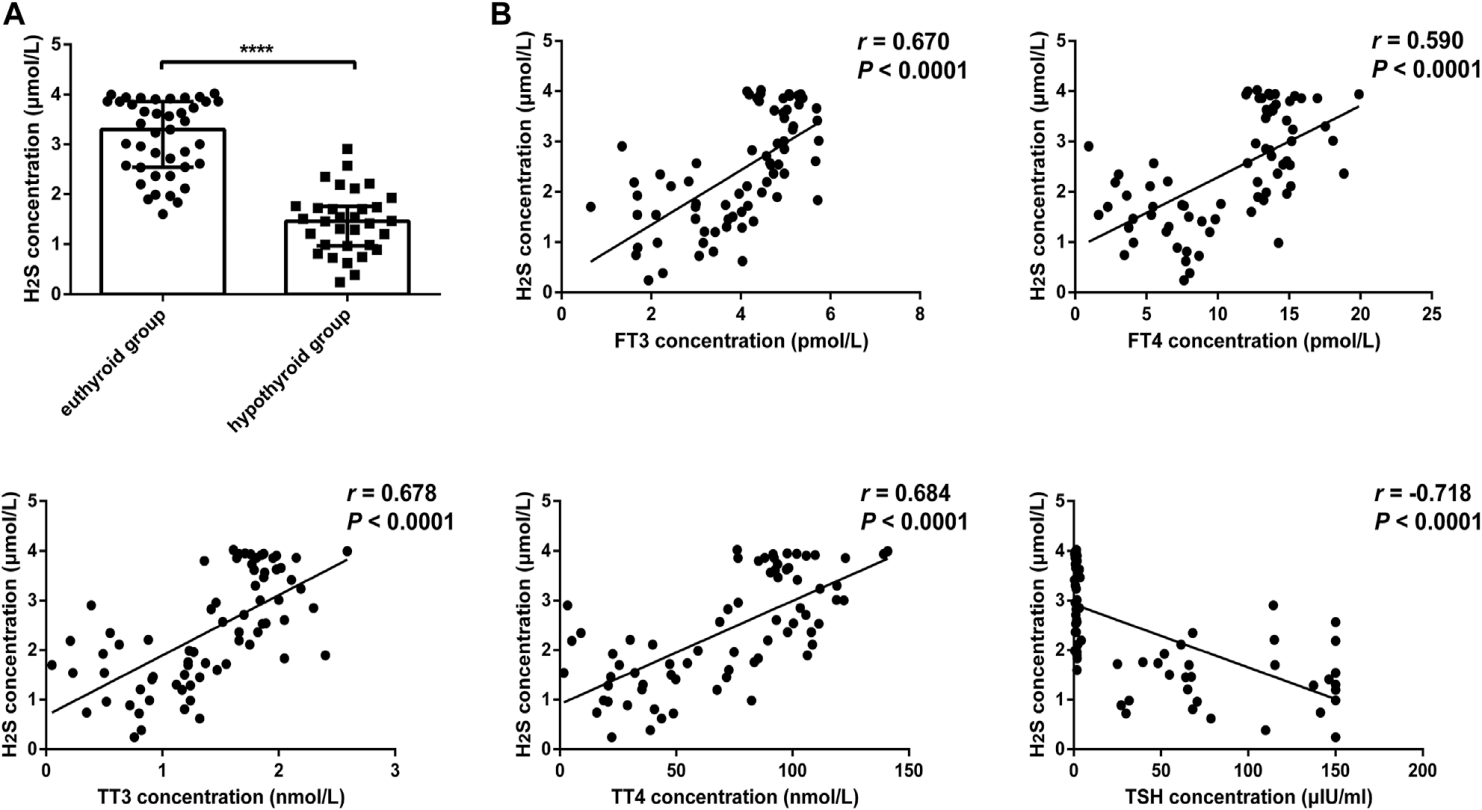
Comparison of H_2_S levels in serum from euthyroid and hypothyroid groups in human and the relationship between H_2_S levels and thyroid function. **(A)** H_2_S levels in serum from the euthyroid (*n* = 41) and hypothyroid (*n* = 32) groups were analyzed by H_2_S -selective sensors. The H_2_S level was significantly lower in the hypothyroid group. **(B)** Correlations between H_2_S levels and thyroid function in serum samples. H_2_S levels were positively correlated with FT3, FT4, TT3, and TT4 levels and negatively correlated with TSH levels. A bivariate correlation analysis was performed by using Spearman rank test. r represents correlation coefficient. ****p < .0001. Data in Figure 2A are expressed as the median and interquartile range. H_2_S, hydrogen sulfide; FT3, free triiodothyronine; FT4, free triiodothyronine; TT3, total triiodothyronine; TT4, total thyroxine; TSH, thyroid-stimulating hormone.

We further compared H_2_S levels between the euthyroid group and hypothyroid group. The clinical features of the subjects are shown in Table 2. The H_2_S levels in serum from the hypothyroid patient group were significantly lower than those in the euthyroid group [1.46 (0.97, 1.76) vs. 3.30 (2.54, 3.86), *p* < .0001] (Figure 2A). As shown in Figure 2B, we observed that the H_2_S level was positively correlated with the serum FT3 level (*r* = 0.670, *p* < .0001), FT4 level (*r* = 0.590, *p* < .0001), TT3 level (*r* = 0.678, *p* < .0001), and TT4 level (*r* = 0.684, *p* < .0001) in all subjects and negatively correlated with the serum TSH level (*r* = −0.718, *p* < .0001). All the results indicated that H_2_S might be associated with thyroid hormone production.

**TABLE 2:** Demographic and clinical characteristics of the euthyroid and hypothyroid groups.

	Euthyroid participants (*n* = 41)	Hypothyroid patients (*n* = 32)	*p* Value
Sex(M/F)	6/35	10/22	.089
Age (years)	46.1 ± 14.5	48.7 ± 20.5	.524
FT3 (pmol/L)	4.9 ± 0.5	2.8 ± 1.0	.0001
FT4 (pmol/L)	13.8 (13.2, 15.1)	6.24 ± 2.9	.0001
TT3 (nmol/L)	1.8 ± 0.3	0.9 ± 0.4	.0001
TT4 (nmol/L)	97.6 ± 17.6	34.4 ± 22.2	.0001
TSH (μIU/ml)	1.5 (0.9, 1.9)	74.7 (56.5, 149.1)	.0001

Data are the mean ± SD, or median and interquartile range. Continuous variables with normal distributions were compared between two groups using independent t tests, and variables with nonnormal distributions were compared between two groups by Mann–Whitney tests. The χ2 test was used to compare categorical variables between the two groups. F, female; M, male; FT3, free triiodothyronine; FT4, free triiodothyronine; TT3, total triiodothyronine; TT4, total thyroxine; and TSH, thyroid-stimulating hormone.

### NaHS Treatment Improved Thyroid Function in Hypothyroid Rats

**FIGURE 3:**
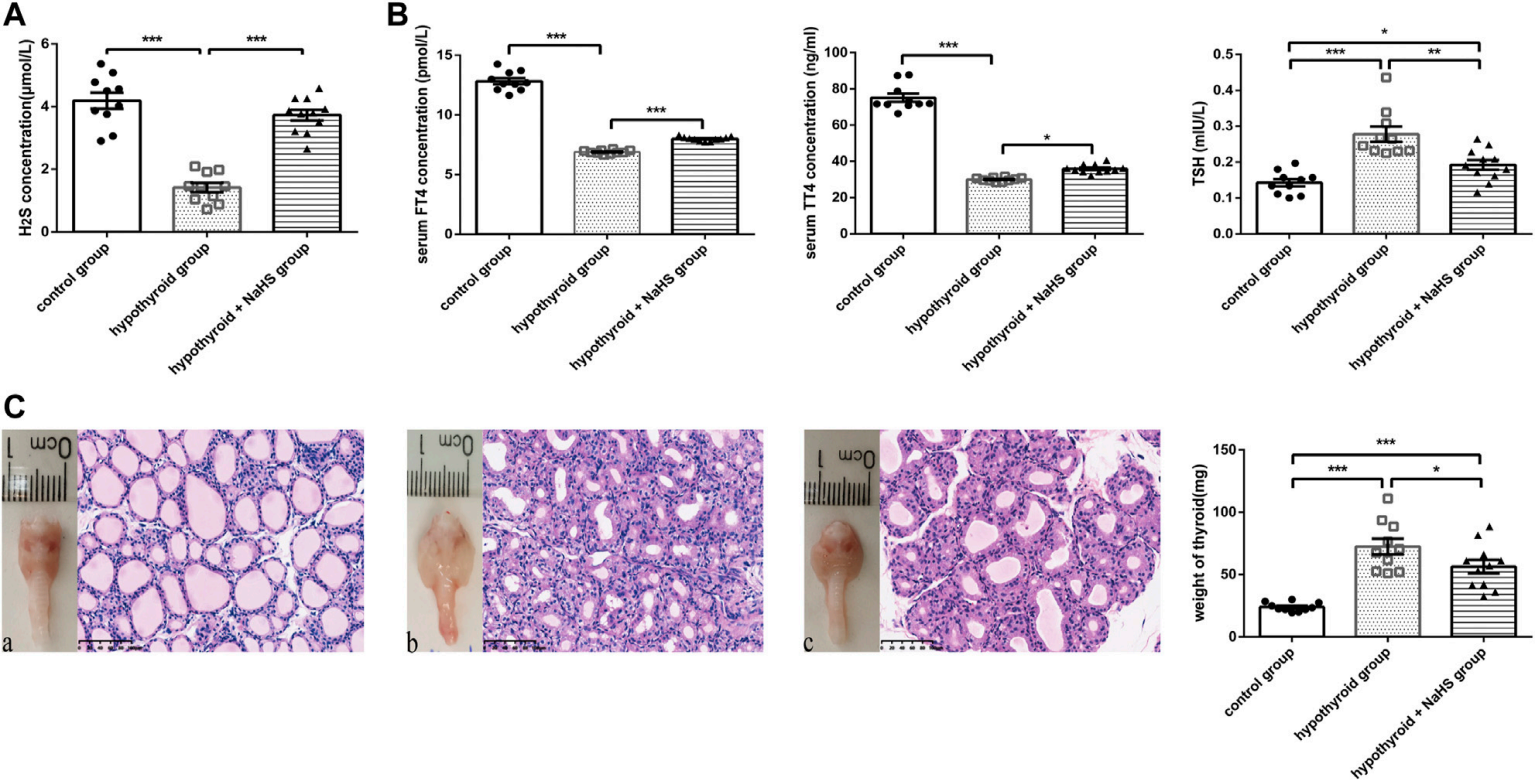
The effect of H_2_S on thyroid function in hypothyroid rats. **(A)** The H_2_S levels in rat serum of control group (*n* = 10), hypothyroid group (*n* = 10) and hypothyroid + NaHS group (*n* = 11) were analyzed by H_2_S-selective sensor. H_2_S level was significantly lower in hypothyroid group and NaHS treatment improved H_2_S level in hypothyroid rats. **(B)** Serum FT4 and TT4 levels in the control group (*n* = 10), hypothyroid group (*n* = 10) and hypothyroid + NaHS group (*n* = 11) were measured by ELISA, and TSH levels were assayed by radioimmunoassay. NaHS treatment improved thyroid function in hypothyroid rats. **(C)** Anterior views of thyroid glands and their related histological changes with H&E staining (magnification, ×200), and the weight of thyroids in different groups are shown. Control group (a), hypothyroid group (b), hypothyroid + NaHS group (c). NaHS treatment alleviated thyroid enlargement in hypothyroid rats. *p < .05, **p < .01, ***p < .001, ****p < .0001. Data are expressed as the mean ± SEM and *n* = 10–11 per group. H_2_S, hydrogen sulfide; NaHS, sodium hydrosulfide; FT4, free triiodothyronine; TT4, total thyroxine; TSH, thyroid-stimulating hormone.

In the hypothyroid model of rats, the H_2_S levels in serum also decreased compared to those in the control group (Figure 3A). After administration of NaHS, the H_2_S level was markedly increased (Figure 3A). H_2_S levels in serum were also positively correlated with serum FT4 levels (*r* = 0.752, *p* < .0001) and TT4 levels (*r* = 0.730, *p* < .0001) and negatively correlated with serum TSH levels (*r* = −0.680, *p* < .0001) (Supplementary Figure S1). This further verified our observation of decreased H_2_S levels in the serum from hypothyroid patients.

To identify the effect of H_2_S on thyroid function, thyroid hormone levels were compared in the control, hypothyroid, and hypothyroid + NaHS groups of Sprague–Dawley rats. Compared with the control group, FT4 and TT4 levels decreased significantly in the hypothyroid group, and TSH levels increased obviously, whereas NaHS treatment clearly reversed the above-described effects in rats (Figure 3B).

Next, to compare the thyroid morphology in the three groups, we performed a gross thyroid inspection in rats and found that the thyroid gland in the hypothyroid group was visually enlarged compared with that in the control group. Meanwhile, thyroid cell hypertrophy and abnormal follicular architecture were also found in the hypothyroid group compared with the control group. The thyroid follicles in the hypothyroid group were lined by hypertrophic and hyperplastic thyrocytes and contained scant colloids. However, these effects in the hypothyroid group were alleviated to a certain extent in the hypothyroid + NaHS group (Figure 3C). This result suggested that NaHS treatment may restore thyroid function and relieve morphological changes of thyrocytes involved in hypothyroidism.

### Effect of H_2_S on Thyroid Hormone Synthesis-Related Molecules and TPO Activity in Human Primary Thyrocytes

**FIGURE 4:**
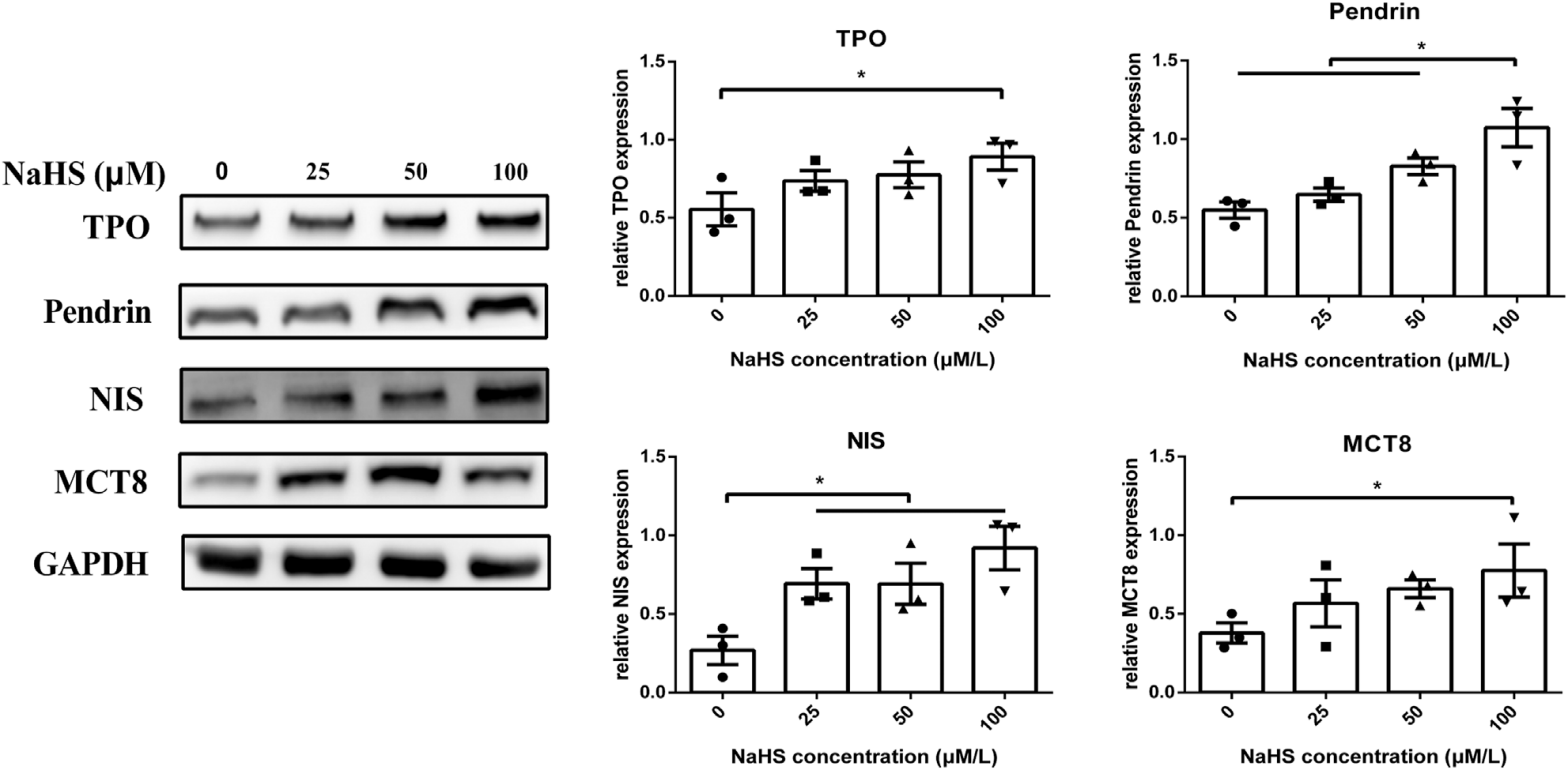
Effect of H_2_S on thyroid hormone synthesis- and secretion-related molecules in human primary thyrocytes. Changes in TPO, Pendrin, NIS, and MCT8 expression in human primary thyrocytes treated with different concentrations of NaHS by Western blot. They were upregulated in a concentration-dependent manner in NaHS.*p < .05, TPO: thyroid peroxidase, NIS: sodium/iodide symporter, MCT8: monocarboxylate transporter eight; NaHS, sodium hydrosulfide. Data are expressed as the mean ± SEM, and all experiments were performed independently three times.

To further explore the potential mechanisms of H_2_S on the synthesis and secretion of thyroid hormones in thyrocytes, we detected synthesis- and secretion-related proteins, such as TPO, NIS, Pendrin and MCT8. As shown in Figure 4, the protein levels of TPO, NIS, Pendrin and MCT8 in human primary thyrocytes were upregulated in a concentration-dependent manner by NaHS.

**FIGURE 5:**
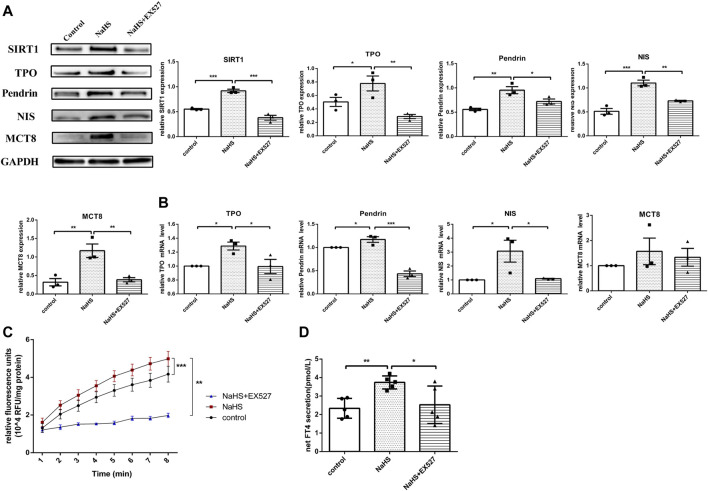
The effect of H_2_S on thyroid hormone and related molecules in human primary thyrocytes. **(A,B)** SIRT1, TPO, Pendrin, NIS, MCT8 protein and mRNA expression in human primary thyrocytes stimulated with NaHS or NaHS + EX527 assessed by real-time PCR and Western blot. **(C)** Extracellular TPO activity of thyrocytes stimulated with NaHS or NaHS + EX527 for 48 h. Fluorescence was normalized to the corresponding protein amounts. **(D)** The FT4 level in supernatants of human primary thyrocytes stimulated with NaHS or NaHS + EX527 for 48 h in chemiluminescence immunoassay. Net FT4 secretion meant that FT4 values in blank culture medium without thyrocytes were subtracted from FT4 content in thyrocyte culture supernatant. *p < 0.05, **p < 0.01, ***p < 0.001. TPO: thyroid peroxidase, NIS: sodium/iodide symporter, MCT8: monocarboxylate transporter eight; NaHS, sodium hydrosulfide. Data are expressed as the mean ± SEM, and all experiments were performed independently at least three times.

Since TPO activity plays an essential role in mediating thyroid hormone synthesis, we compared TPO activity in human primary thyrocytes treated with or without NaHS. We found that TPO activity was upregulated when thyrocytes were challenged with NaHS (Figure 5C).

### H_2_S Promoted Thyroid Hormone Synthesis and Secretion by Increasing the Expression of Related Proteins and TPO Activity via SIRT1 in Human Primary Thyrocytes

H_2_S is involved in some physio-pathological processes by regulating SIRT1 (Xin et al., 2016; Guan et al., 2020). To investigate whether H_2_S exerted functions on regulating TPO, NIS, Pendrin and MCT8 expression through SIRT1, human primary thyrocytes were divided into control, NaHS or NaHS + EX527 groups. After stimulating with NaHS, both the mRNA and protein expression levels of TPO, NIS and Pendrin were upregulated, and MCT8 was upregulated at the protein level (Figures 5A,B). However, pretreating thyrocytes with EX527 clearly reversed the above-described effects (Figures 5A,B).

We subsequently identified whether H_2_S promoted TPO activity by regulating SIRT1 in human primary thyrocytes. As shown in Figure 5C, the increased extracellular TPO activity in thyrocytes after incubation with NaHS was suppressed by EX527.

Accordingly, the FT4 level in the human primary thyroid cell supernatant was increased after incubation with NaHS, and after blocking SIRT1 with EX527, the FT4 level was decreased (Figure 5D).

## Discussion

Hypothyroidism is an endocrinopathy caused by thyroid hormone production and secretion deficiency in the thyroid gland. The mechanism of hypothyroidism involves the destruction of thyroid cell functions that synthesize thyroid hormones (Gillam and Kopp, 2001). In our study, we firstly demonstrated that H_2_S, a gas signaling molecule, might have a protective effect on thyroid hormone synthesis and secretion by promoting the expression levels of thyroid hormone synthesis-related proteins and upregulating TPO activity through SIRT1.

H_2_S is produced in mammals by three major H_2_S-generating enzymes: CBS, CSE, and 3-MPST (Braunstein et al., 1969; Chiku et al., 2009; Shibuya et al., 2009). CBS appears abundantly in the central nervous system, and CSE is mainly found in cardiovascular tissues (Pan et al., 2012). In our research, we found that H_2_S production in thyrocytes, and CBS, CSE, and 3-MPST all existed in thyrocytes, which indicated that H_2_S was automatically generated by thyroid cells. According to the difference in mRNA expression, CBS was the main H_2_S-generating enzyme in the thyroid, further illustrating that the expression of H_2_S-generating enzymes was tissue specific.

There have been extensive reports about the protective effect of H_2_S on various diseases by diverse S-sulfhydration molecules. Tian et al. reported that H_2_S inhibited the proliferation of vascular smooth muscle cells by persulfidating FOXO1 at Cys457 to protect vascular structure, demonstrating that H_2_S had a beneficial effect on cardiovascular diseases (Tian et al., 2021). H_2_S levels were decreased in ischemic vascular dementia, and NaHS protected against neuronal injury in a vascular dementia rat model (Zhang et al., 2009). Du et al. also proposed that CSE/H_2_S promoted the deacetylation activity of SIRT1 by sulfhydrating SIRT1 and thus has an antiatherogenetic role (Du et al., 2019). In our research, we found that serum H_2_S levels were decreased in both hypothyroid patients and rats. Furthermore, FT4 and TT4 levels were improved by NaHS treatment in hypothyroid rats. These results revealed that H_2_S had a role in protecting thyroid functions.

Subsequently, we studied how H_2_S might influence thyroid functions. Mechanisms involved in thyroid hormone synthesis and secretion include oxidation of the TPO, iodination reaction, coupling reaction, iodide recycling and thyroid hormone transport (Carvalho and Dupuy, 2017). TPO and TPO activity, Pendrin, NIS, and MCT8 were essential in this process. *In vitro*, we determined that the expression of TPO, Pendrin, NIS, and MCT8 was upregulated in a concentration-dependent manner in thyrocytes incubated with NaHS. TPO activity was also increased under NaHS treatment. These changes were consistent with the increase in FT4 levels. This result indicated that H_2_S increased the synthesis and secretion of thyroid hormones in thyrocytes by upregulating the expression of associated molecules and TPO activity.

However, how H_2_S induces this effect is not yet known. In the literature, Chen et al. showed that high glucose triggered reduced expression of thyroid hormone synthesis- and secretion-related proteins, which was accompanied by SIRT1 downregulation (Chen et al., 2019). We found that H_2_S promoted the expression of TPO, NIS, Pendrin, MCT8 and TPO activity through SIRT1, which resulted in an increase in FT4 levels. When thyrocytes were incubated with EX527 to inhibit SIRT1, the above regulatory effect of NaHS disappeared. We proposed that H_2_S promoted thyroid hormone synthesis and secretion by upregulating SIRT1.

Alleviated secretion of thyroid hormones by thyroid cells is one of the mechanisms of hypothyroidism (Gillam and Kopp, 2001). In our study, we demonstrated that H_2_S improved thyroid hormone levels in hypothyroid rats and promoted the expression of thyroid hormone synthesis and secretion and TPO activity in human primary thyrocytes. H_2_S, as a gaseous signaling molecule, has been shown to be involved in multiple physiological and pathophysiological processes. Several H_2_S-based therapy drugs have been developed to preclinical and early clinical stages, such as AP39 (Szczesny et al., 2014) and SG-1002 (Elrod et al., 2007). According to these developments, a better understanding of the H_2_S mechanism in different organs will be necessary (Wallace and Wang, 2015). We found that H_2_S has a protective effect on hypothyroidism; thus, H_2_S-related drugs may become an alternative treatment for hypothyroidism.

Our study had some limitation. Firstly, we found that H_2_S promoted thyroid hormone synthesis and secretion by increasing the expression of related proteins in human primary thyrocytes. However, thyroid tissue specimens from rats were limited, and we did not explore whether H_2_S regulated synthesis- and secretion-related molecules to restore thyroid hormones in hypothyroid rats. Secondly, we demonstrated that H_2_S levels were reduced in hypothyroid patients and had a protective role in thyroid function, but we did not investigate the mechanism of the decrease in H_2_S levels in thyrocytes. It was reported that the levels of CBS, CSE, and 3-MPST were changed in the muscles and liver tissues of hyperthyroid rats in different ways (Jeddi et al., 2019), which indicated that thyroid hormone influenced the expression of H_2_S-generating enzymes in rats. Further research will be performed to test whether the decreased H_2_S level was induced by a hypothyroid status.

In conclusion, we confirmed that human thyrocytes produce H_2_S, and we found that CBS is the main H_2_S-generating enzyme. H_2_S enhances the expression of thyroid hormone synthesis- and secretion-related molecules and TPO activity, which in turn promotes the synthesis and secretion of thyroid hormones by upregulating SIRT1. Our work has shown that H_2_S participates in the synthesis and secretion of thyroid hormones, which may provide a new view of H_2_S for treating hypothyroidism.

## Data Availability

The original contributions presented in the study are included in the article/Supplementary Material further inquiries can be directed to the corresponding author.
