# Supplementary material for: Hydrogen Sulfide Promotes Thyroid Hormone Synthesis and Secretion by Upregulating Sirtuin-1
Source: Front Pharmacol. 2022 Feb 10;13:838248. doi: 10.3389/fphar.2022.838248 (PMC8866871; doi:10.3389/fphar.2022.838248)
Supplement: Supplementary file 1 [file Image1.pdf]

Supplementary Material

1 Supplementary Figures

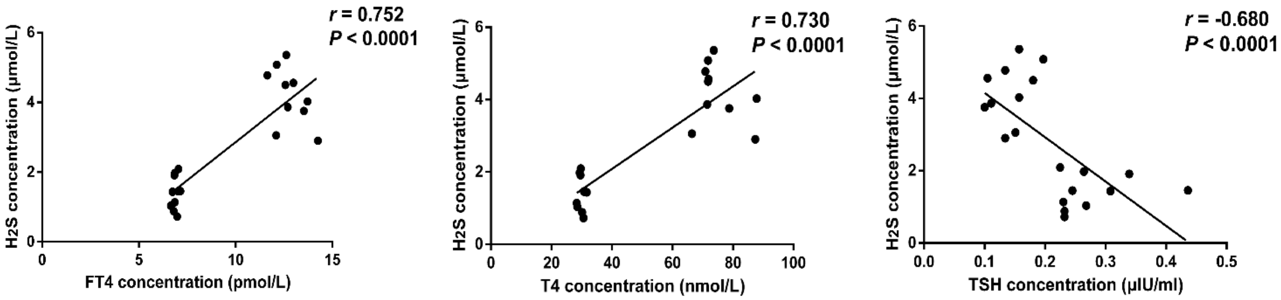

Figure S1. Correlations between the serum H_2S level and serum FT4, TT4 and TSH levels in control and hypothyroid group of Sprague–Dawley rats. H_2S levels were positively correlated with FT4 and TT4 levels and negatively correlated with TSH levels.

A bivariate correlation analysis was performed by using the Spearman rank test. r represents the correlation coefficient.H_2S, hydrogen sulfide; FT4, free triiodothyronine; TT4, total thyroxine; and TSH, thyroid-stimulating hormone.
